# Neurocognitive Sequelae of COVID‐19 in Older African American Adults

**DOI:** 10.1002/alz.089637

**Published:** 2025-01-03

**Authors:** Tiffany A Walker, Jannah Elchommali, Jenny E Han, Alexander Truong, Ihab Hajjar, Felicia Goldstein

**Affiliations:** ^1^ Emory University, Atlanta, GA USA; ^2^ Grady Memorial Hospital, Atlanta, GA USA; ^3^ UT Southwestern Medical Center, Dallas, TX USA; ^4^ Emory University School of Medicine, Atlanta, GA USA

## Abstract

**Background:**

African Americans (AA) are disproportionately affected by Long COVID, highlighting the need for targeted research to understand the enduring consequences of COVID‐19 within this community. Among the array of symptoms associated with post‐acute sequelae of SARS‐CoV‐2 (PASC), cognitive impairments emerge as a significant concern affecting up to 19% of COVID survivors. In this study, our goal is to comprehensively characterize the specific cognitive domains impacted in older AA adults.

**Method:**

This prospective, cohort study collected sociodemographic data and conducted a telephone‐based neurocognitive battery on AA adults aged ≥ 50 years with persistent symptoms ≥ 12 months following confirmed SARS‐CoV‐2 infection, measuring cognitive domains of global function (Blind Montreal Cognitive Assessment (MoCA)), attention (Blind MoCA Attention subscale, Number Span Forward), memory (Hopkins Verbal Learning Test (HVLT), Craft Story 21)), and executive function (Number Span Backward). Z‐scores were calculated based on the means and SDs of demographically comparable normative samples. Impaired performance was defined as z scores > ‐1.5.

**Result:**

We enrolled 75 AAs with a median age of 58 years (range 51‐73); 65 (86%) were female, and 69 (92%) had a medium‐high social vulnerability index. Forty (53%) participants exhibited global impairment on Blind MOCA. 54 (72%) had impairment on ≥ 1 attention test, including 53 (70%) with impaired MoCA Attention subscale scores and 5 (6%) with impaired Number Span Forward testing. Fifty‐three (70%) had impairment on ≥ 1 memory test, including 42‐48% with impaired HVLT and 45‐48% with impaired Craft Story 21. Four (5%) had impaired executive function on Number Span Backwards testing.

**Conclusion:**

We demonstrate persistent COVID‐19 cognitive impairments impacting memory, attention, executive function, and global function within a socioeconomically disadvantaged older AA adult population. Limited access to healthcare resources in medically underserved areas can lead to delayed or inadequate COVID‐19 treatment, potentially increasing the risk of neurological complications and cognitive deficits in African American populations.

